# Tratamiento para tuberculosis resistente a rifampicina o multirresistente: análisis comparativo de indicadores programáticos entre Buenaventura y otros municipios del Valle del Cauca, Colombia

**DOI:** 10.7705/biomedica.7204

**Published:** 2024-08-29

**Authors:** Diana Hoyos, Rossi Meza, Liliana Forero, César Moreira, Beatriz E. Ferro, Robinson Pacheco

**Affiliations:** 1 Secretaría de Salud del Valle del Cauca, Cali, Colombia Secretaría de Salud del Valle del Cauca Secretaría de Salud del Valle del Cauca Cali Colombia; 2 Berlin School of Public Health, Charité Universitâtsmedizin, Berlin, Alemania Secretaría de Salud del Valle del Cauca Berlin School of Public Health Berlin Germany; 3 Facultad de Ciencias de la Salud, Universidad Icesi, Cali, Colombia Universidad Icesi Universidad Icesi Cali Colombia; 4 Grupo Interdisciplinario de Investigación en Epidemiología y Salud Pública, Universidad Libre, Cali, Colombia Universidad Libre Universidad Libre Cali Colombia

**Keywords:** Mycobacterium tuberculosis, tuberculosis, tuberculosis resistente a múltiples medicamentos, terapéutica, programa, efectos colaterales y reacciones adversas relacionados con medicamentos, Colombia, Mycobacterium tuberculosis, tuberculosis, tuberculosis, multidrug-resistant, therapeutics, program, drug-related side effects and adverse reactions, Colombia

## Abstract

**Introducción.:**

El manejo adecuado de la tuberculosis multirresistente es una estrategia priorizada para el control de la tuberculosis en el mundo.

**Objetivo.:**

Evaluar las diferencias entre las características demográficas y clínicas, y los indicadores programáticos de los pacientes con diagnóstico confirmado de tuberculosis pulmonar resistente a rifampicina o multirresistente en Buenaventura, frente a la cohorte de los demás municipios del Valle del Cauca entre 2013 y 2016.

**Materiales y métodos.:**

Se desarrolló un estudio analítico de cohortes para comparar los registros de pacientes mayores de 15 años con tuberculosis multirresistente, del Programa de Tuberculosis de Buenaventura (con ácido paraaminosalicílico), frente a los demás municipios del Valle del Cauca (sin ácido paraaminosalicílico).

**Resultados.:**

Se registraron 99 casos con una mediana de edad de 40 años (RIC = 26 - 53); en Buenaventura, el 56 % eran mujeres; en los demás municipios, predominaron los hombres (67 %); el 95 % de los evaluados tenía aseguramiento en salud. La comorbilidad más frecuente fue diabetes (14 %). Las reacciones adversas a medicamentos antituberculosos en Buenaventura fueron 1,3 veces más frecuentes que en los demás municipios (OR = 2,3; IC_95 %_: 0,993 - 5,568; p = 0,04). En Buenaventura falleció el 5 % de los casos frente al 15 % reportado en los demás municipios. No hubo fracasos con el tratamiento en Buenaventura, pero se reportó un 35 % de pérdida del seguimiento. El éxito del tratamiento fue mayor en Buenaventura en el 56 %.

**Conclusión.:**

El programa fortalecido de Buenaventura presentó mejores resultados programáticos que los demás municipios del Valle del Cauca. El acceso a pruebas moleculares, la disponibilidad de tratamientos acortados y el seguimiento continuo para identificar reacciones adversas a medicamentos antituberculosos son un derrotero para todos los programas de control.

## Abstract (en)

**Introduction.:**

Proper management of multidrug-resistant tuberculosis is a prioritized strategy for tuberculosis control worldwide.

**Objective.:**

To evaluate differences concerning demographic and clinical characteristics and programmatic indicators of Buenaventura patient cohort with confirmed diagnosis of multidrug-resistant tuberculosis, compared to those of the other municipalities from Valle del Cauca, Colombia, 2013-2016.

**Materials and methods.:**

We conducted an analytical cohort study to compare records of patients older than 15 years with multidrug-resistant tuberculosis included in the *Programa de Tuberculosis de Buenaventura* (with para-aminosalicylic acid) versus the other municipalities of Valle del Cauca (without para-aminosalicylic).

**Results.:**

Ninety-nine cases were recorded with a median age of 40 years (IQR = 26 - 53); in Buenaventura, 56% of the patients were women, while in the other municipalities, men predominated with 67%; 95% had health insurance. The most common comorbidity was diabetes (14%). Adverse reactions to antituberculosis medications in Buenaventura were 1.3 times more frequent than in the other municipalities (OR = 2.3; 95% CI = 0.993 - 5.568; p = 0.04). In Buenaventura, the mortality rate was 5% compared to the 15% reported in the other municipalities. Treatment failures were not reported in Buenaventura, but 35% did not continue with the follow-up. Treatment success was higher in Buenaventura (56 %).

**Conclusion.:**

A strengthened program in Buenaventura presented better programmatic results than those from the other municipalities of Valle del Cauca. Access to molecular tests, availability of shortened treatments, and continuous monitoring to identify adverse reactions to antituberculosis medications are routes for all other control programs.

La detección temprana y el tratamiento oportuno de los casos de tuberculosis multirresistente (*multidrug-resistant*, MDR) son algunas de las actividades priorizadas por las estrategias para el control de la tuberculosis en el mundo ^1^. La Organización Mundial de la Salud (OMS) estima que anualmente el 3,3 % de los casos nuevos de tuberculosis y el 18 % de los previamente tratados, reportan resistencia a rifampicina (RR) o son resistentes a rifampicina e isoniacida (MDR), lo que representa alrededor de 500.000 casos en el mundo. Infortunadamente, solo la tercera parte accede al tratamiento, con un éxito terapéutico del 59 % ^2^^,^^3^. En Colombia, el 2,4 % de los casos nuevos y el 14 % de los previamente tratados son de tuberculosis MDR y el éxito terapéutico es del 45 %. El departamento del Valle del Cauca aportó el 25 % de los casos farmacorresistentes en el 2019 y el 14 % en el 2020 ^4^, mientras que el puerto de Buenaventura aportó el 6 % de los casos nuevos, siendo uno de los porcentajes más altos de Latinoamérica ^5^.

Hasta el 2020, las guías colombianas de atención recomendaban practicar pruebas de sensibilidad a fármacos prioritariamente en la población de mayor riesgo. En los casos de tuberculosis RR o MDR, se sugería el inicio del tratamiento estandarizado de categoría IV que incluye kanamicina, levofloxacina, etionamida, cicloserina, etambutol y pirazinamida, y de esquemas individualizados una vez disponibles las pruebas de sensibilidad ^6^.

En Buenaventura, el esquema fue ajustado al perfil de resistencia del municipio, reemplazando la kanamicina por capreomicina y la levofloxacina por moxifloxacina, y adicionando ácido paraaminosalicílico (PAS) según la recomendación del Comité de Evaluación Regional de Casos Especiales de Tuberculosis (CERCET, 2011), para procurar un mayor éxito terapéutico ^7^.

Teniendo en cuenta que la administración de un mayor número de medicamentos, además de representar mayores costos, puede aumentar la toxicidad, reducir el cumplimiento del tratamiento y afectar los resultados programáticos, el objetivo de esta investigación fue evaluar las diferencias entre las características demográficas y clínicas, y los indicadores programáticos de los pacientes con diagnóstico confirmado de tuberculosis pulmonar RR o MDR de Buenaventura, frente a los de la cohorte de los demás municipios del Valle del Cauca entre el 2013 y el 2016.

## Materiales y métodos

Se llevó a cabo un estudio analítico de cohortes en el que se compararon variables sociodemográficas, clínicas y programáticas de pacientes mayores de 15 años con tuberculosis RR o MDR del Programa de Tuberculosis de Buenaventura, frente a aquellos de los demás municipios del departamento del Valle del Cauca.

### Población de estudio

La población de estudio estuvo conformada por los pacientes con tuberculosis cuyo perfil de resistencia correspondía a RR o MDR, registrados en el Programa de Control de Tuberculosis del departamento del Valle del Cauca.

### Criterios de selección

Se analizó la información de los registros que cumplieron los criterios de inclusión de ser pacientes con tuberculosis pulmonar confirmada, con edad de 15 o más años, a quienes se les confirmó el perfil de resistencia por prueba fenotípica o molecular: GenoType MTBDR plus para rifampicina e isoniacida (perfil MDR), o GeneXpert MTB/RIF para resistencia a rifampicina (perfil RR), y quienes iniciaron el tratamiento en categoría IV entre el 2013 y el 2016.

Si bien las dos pruebas mencionadas no generan resultados iguales, las limitaciones de acceso a otras pruebas durante el tiempo de referencia de este estudio impedía la confirmación de los perfiles y, para los programas, el perfil RR se aproximaba al de MDR para su manejo. Se excluyeron aquellos registros que presentaron diferente patrón de resistencia a RR o MDR, los que no tuvieran confirmación bacteriológica de la resistencia y los que no tuvieran información sobre el seguimiento.

### Variables y fuentes de información

La información sobre edad, sexo, régimen de afiliación, comorbilidades, coinfección de tuberculosis y HIV, uso de PAS, reacciones adversas a fármacos antituberculosos (RAFAS), condición de ingreso, presentación al CERCET, tipo de resistencia, métodos diagnósticos y resultados programáticos, fue extraída de las siguientes fuentes de información: “Libro de pacientes farmacorresistentes del Valle del Cauca”, historias clínicas, tarjetas individuales de tratamiento, reportes del Sistema de Vigilancia en Salud Pública (SIVIGILA), actas del CERCET y bases de datos del Laboratorio Departamental de Salud Pública del Valle del Cauca, facilitadas por el Programa de Tuberculosis Departamental del Valle del Cauca.

Los resultados programáticos considerados fueron: “fallecido”, paciente afectado por tuberculosis que fallece por cualquier razón antes de iniciar el tratamiento o durante el mismo; “pérdida de seguimiento”, paciente afectado por tuberculosis que no inició tratamiento o interrumpió el tratamiento durante un mes o más; “curado”, paciente con tratamiento completo sin evidencia de fracaso y tres o más cultivos negativos con intervalo de 30 días en los últimos meses de tratamiento; y “tratamiento terminado”, paciente con tratamiento completo sin evidencia de fracaso, pero sin constancia de cultivos negativos consecutivos en los últimos meses de tratamiento.

### Análisis estadístico

La información fue recolectada en Microsoft Office Excel^®^ 2016 y analizada con Stata 12^TM^ (Stata Corp, College Station, TX, USA). Las características clínicas, demográficas y programáticas se resumieron mediante estadística descriptiva; las variables numéricas se representaron con la mediana y los rangos intercuartílicos (RIC) y las cualitativas se presentaron como proporciones en tablas de frecuencias.

Para evaluar diferencias respecto a las características demográficas y los indicadores programáticos entre la cohorte de Buenaventura y la de los demás municipios del Valle del Cauca, se aplicaron pruebas estadísticas como ji al cuadrado (c^2^) para comparar proporciones y la prueba de Mann- Whitney para comparar medianas. La significancia estadística se asumió con valores de p inferiores a 0,05. Finalmente, para determinar la magnitud y la dirección de la fuerza de asociación, se usó la razón de probabilidades (OR) con sus respetivos intervalos de confianza del 95 %.

Se usó el OR por ser una medida de asociación más versátil que el riesgo relativo y que puede usarse, con mucha prudencia en su interpretación, en cualquier diseño, aun en los transversales y los experimentales; además, los eventos evaluados presentan una frecuencia menor del 10 %, situación en la que las dos medidas dan resultados similares. Otra ventaja del uso del OR en este diseño analítico de cohortes es que, de ser necesario un ajuste multivariado, puede modelarse el OR mediante una regresión.

### Consideraciones éticas

El estudio fue avalado como una investigación sin riesgo por el Comité de Ética de la Universidad Libre (acta 006-2019).

## Resultados

**Figura 1:**
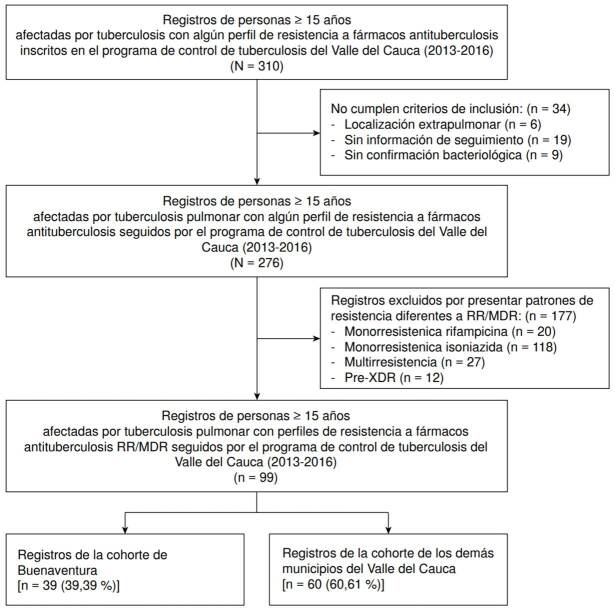
Diagrama de flujo de pacientes con diagnóstico de tuberculosis farmacorresistente registrados en el programa de control de tuberculosis del Valle del Cauca durante 2013 a 2016 y que recibieron tratamiento estandarizado para tuberculosis resistente a rifampicina o multirresistente y su historia de tratamiento

Durante el período de estudio, se evidenció algún tipo de resistencia en 310 registros, pero solo 276 fueron incluidos. Los demás no ingresaron por ser de pacientes con enfermedad extrapulmonar (n = 6; 2,6 %), no tener información de seguimiento (n = 19; 9,0 %) o no tener confirmación bacteriológica (n = 9; 4,3 %), lo cual reduciría la calidad de los datos. De los 276 incluidos, 177 registros fueron excluidos por las siguientes razones: resistencia a isoniacida (n = 118; 55,9 %), resistencia a rifampicina (n = 20; 9,5 %), multirresistencia (n = 27; 12,8 %) y casos extremadamente resistentes (*extensively drug-resistant*, pre-XDR (12; 5,7%). En total, quedaron 99 registros para el análisis: 39 (39,4%) correspondientes a pacientes del programa de Buenaventura y, 60 (60,6 %), a los demás municipios del Valle del Cauca: 46 de Cali, 6 de Palmira, 2 de Tuluá y 1 de Cartago, Guacarí, El Cerrito, Jamundí, Restrepo y Yumbo (figura 1).

*Condición de ingreso nuevo: pacientes sin tratamiento previo para tuberculosis o que lo recibieron por menos de 30 días.

**Figura 2:**
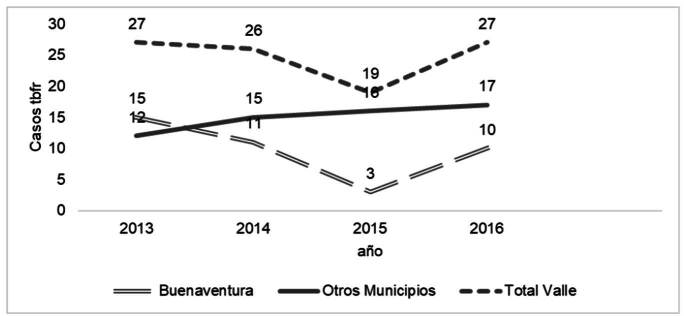
Tendencia de casos de tuberculosis resistente a rifampicina (RR) o multirresistente (MDR) de Buenaventura y otros municipios del departamento del Valle del Cauca, notificados en el Sistema Nacional de Vigilancia Epidemiológica (SIVIGILA) entre el 2013 y el 2016 (n = 99)

El comportamiento anual de los casos de RR o MDR fue estable, excepto en 2015 cuando hubo un menor número de casos en la cohorte de Buenaventura, con una ligera tendencia al aumento en la cohorte de los demás municipios (figura 2).

Las características demográficas y clínicas mostraron que la mediana de edad fue de 40 años (RIC = 26-53), sin diferencias significativas entre Buenaventura (mediana = 42 años; RIC = 32-51) y los demás municipios (mediana = 37 años, RIC = 25-53). Los casos de mujeres predominaron en Buenaventura (22/39; 56 %) y los de hombres (40/60; 67 %) en los demás municipios. El 5 % de los pacientes no tenía seguro médico.

En el 45 % (45/99) de los registros se documentó, al menos, una comorbilidad, siendo diabetes la más frecuente con el 14 % (14/99), seguida de desnutrición con el 11 % (11/99); Buenaventura aportó seis casos de diabetes y tres de desnutrición; los demás municipios aportaron ocho casos de diabetes y desnutrición, respectivamente.

De la totalidad de la cohorte, 94 pacientes recibieron asesoría y prueba voluntaria para detección de HIV, y cinco ya presentaban diagnóstico previo. En total, ocho personas con HIV (8 %) fueron incluidas en las cohortes (tres en Buenaventura, cinco en los demás municipios: tres de Cali y dos de Palmira), de los cuales tres fueron diagnosticados de novo con dicha condición; solo de uno de ellos no se obtuvo información sobre la administración de terapia antirretroviral (cuadro 1).

**Cuadro 1:** Características demográficas y clínicas extraídas de los registros de los pacientes incluidos en los esquemas de tratamiento para tuberculosis resistente a rifampicina o multirresistente en el Valle del Cauca, Colombia, 2013-2016 (N = 99)

Característica	Descripción	n	Buenaventura (n = 39)	Demás municipios (n = 60)	p
Edad (años)	Mediana	40	42	37	0,492
	Rango intercuartílico	26 - 53	32 - 51	25 - 53	
Sexo [n (%)]	Masculino	57	17 (43,58)	40 (66,66)	0,023
	Femenino	42	22 (56,41)	20 (33,33)	
Afiliación [n (%)]	Asegurado	94	37 (94,87)	57 (95,00	0,977
	No Asegurado	5	2 (5,12)	3 (5,00)	
Comorbilidades [n (%)]	Sí	45	14 (35,89)	31 (51,66)	0,124
	No	54	25 (64,10)	29 (48,33)	
Tipos de comorbilidades [n (%)]	Diabetes	14	6 (42,85)	8 (57,14)	0,775
	Desnutrición	11	3 (27,27)	8 (72,73)	0,388
	HIV	8	3 (37,50)	5 (62,50)	0,909
	Otras	12	2 (16,66)	10 (83,34)	0,104
Coinfección TB/HIV [n (%)]	Negativo	91	36 (92,30)	55 (91,66)	0,385
	Positivo	8	3 (7,69)	5 (8,33)	
Condición de ingreso [n (%)]	Nuevo	56	27 (69,23)	29 (48,33)	0,040
	Previamente tratado	43	12 (30,76)	31 (51,66)	
Asesoría y prueba voluntaria de HIV [n (%)]	Sí	94	39 (100)	55 (91,66)	0,064
	No	5	0	5 (8,33)	
Tipo de resistencia [n (%)]	MDR	80	28 (71,79)	52 (86,66)	0,066
	RR	19	11 (28,20)	8 (13,33)	
Método diagnóstico de resistencia [n (%)]	Con BACTEC MGIT	17	5 (12,82)	12 (20,00)	0,355
	Sin BACTEC MGIT	82	34 (87,17)	48 (80,00)	
	Con LiPA (GenoType MTBDR plus)	36	2 (5,12)	34 (56,66)	0,000
	Sin LiPA (GenoType MTBDR plus)	63	37 (94,87)	26 (43,33)	
	Con real qPCR (GeneXpert MTB/RIF)	46	32 (82,05)	14 (23,33)	<0,000
	Sin qPCR (GeneXpert MTB/RIF)	53	7 (17,94)	46 (76,66)	

BACTEC MGIT: método de cultivo no reactivo automatizado, sensibilidad de 100 % para los cuatro medicamentos (isoniacida, rifampicina, etambutol y estreptomicina) y un rango de especificidad de 89,8 % para estreptomicina y hasta de 100 % para rifampicina.

LiPA: prueba molecular basada en la tecnología de sondas en línea. Permite la identificación genética del complejo Mycobacterium tuberculosis y de las mutaciones de los genes *rpoB*, *katG* e *inhA* que confieren resistencia a rifampicina e isoniacida.

qPCR: técnica molecular que permite la detección de *Mycobacterium tuberculosis* en muestras pulmonares mediante la amplificación de genes específicos y mutaciones más frecuentes en el gen *rpoB* para demostrar resistencia a rifampicina

Respecto a los indicadores programáticos, según la condición de ingreso, Buenaventura reportó una mayor proporción de casos nuevos (27/39; 69 %) en comparación con los tratados, mientras que, en el resto del departamento, la proporción de casos nuevos y previamente tratados fue similar (29/60; 51 %). Con referencia al método diagnóstico usado para detectar la resistencia, en 32 de los 39 casos registrados en Buenaventura se usó la prueba GeneXpert MTB/RIF (82 %) con la cual se identificó el perfil de tuberculosis RR. En los demás municipios, el método de detección de resistencia más frecuente fue GenoType MTBDR plus (34/60; 57 %), con el cual se identificó el perfil de tuberculosis MDR. El tiempo promedio transcurrido desde el diagnóstico hasta el inicio del tratamiento fue de 37 días en Buenaventura y de 72 días en los demás municipios.

Se evidenciaron diferencias respecto a la frecuencia de RAFAS: en Buenaventura fueron más frecuentes que en los demás municipios (OR = 2,3 IC_95%_: 0,993 - 5,568; p = 0,04). Otras variables, como la valoración por el CERCET (OR = 1,952; IC_95%_: 0,588 - 7,606; p = 0.275) y el reajuste del esquema de medicamentos antituberculosos (OR = 2,071; IC_95%_: 0,865 - 5,005; p = 0.093), no fueron estadísticamente significativas; tampoco hubo diferencias en la duración del tratamiento (mediana = 452,69 días para Buenaventura *versus* 416,11 días para los demás municipios; p = 0,2523). Sin embargo, 30 de los 31 pacientes que tuvieron ajustes en el tratamiento, fueron presentados al CERCET y, de estos, 16 (53 %) eran de Buenaventura (cuadro 2).

**Cuadro 2:** Diferencias programáticas de los esquemas de tratamiento para tuberculosis resistente a rifampicina o multirresistente en el Valle del Cauca, Colombia, 2013-2016

Característica	Descripción	n	Buenaventura (n = 39)	Otros municipios (n = 60)	OR	IC_95_%	p
Tiempo promedio de inicio de	Diferencia en días entre	63	37	72	-	-	0,118
tratamiento	inicio de tratamiento y fecha de diagnóstico DE	120,6	73,2	142,1			
Presentado en CERCET [n (%)]	Sí	84	35 (89,74)	49 (81,66)	1,952	0,5879; 7,606	0,275
	No	15	4 (10,25)	11 (18,33)			
RAFAS [n (%)]	Sí	34	18 (46,15)	16 (26,66)	2,336	0,9933; 5,568	0,046
	No	65	21 (53,84)	44 (73,33)			
Reajuste tratamiento [n (%)]	Sí	31	16 (41,02)	15 (25,00)	2,071	0,8653; 5,005	0,093
	No	68	23 (58,97)	45 (75,00)			
Días en tratamiento	Mediana (RIC)	430,5	452,69	416.11	-	-	0,252
		(178 - 628)	(156 - 645)	(186 - 600,5)			
Fallecido [n (%)]	Sí	11	2 (5,12)	9 (15,00)	3,264	0,6167; 32,455	0,127
	No	88	37 (94,87)	51 (85,00)			
Pérdida del seguimiento [n (%)]	Sí	37	15 (38,46)	22 (35,00)	0,926	0,3455; 2,1692	0,857
	No	62	24 (61,53)	38 (65,00)			
Curado [n (%)]	Sí	40	18 (46,15)	22 (36,67)	0,6754	0,2750; 1,6636	0,347
	No	59	21 (53,84)	38 (63,33)			
Tratamiento terminado [n (%)]	Sí	11	4 (10,25)	7 (11,67)	11,556	0,2693; 5,785	0,827
	No	88	35 (89,74)	53 (88,33)			

CERCET: Comité de Evaluación Regional de Casos Especiales de Tuberculosis; RAFAS: reacciones adversas a fármacos antituberculosos; RIC: rango intercuartílico; DE: desviación estandar

Condiciones de egreso:

* Fallecido: persona afectada por tuberculosis que fallece por cualquier razón antes de iniciar el tratamiento o durante el tratamiento.

** Pérdida de seguimiento: persona afectada por tuberculosis que no inició tratamiento o interrumpió el tratamiento durante un mes o más.

*** Curado: persona con tratamiento completo, sin evidencia de fracaso y tres o más cultivos negativos con intervalo de 30 días en los últimos meses de tratamiento.

**** Tratamiento terminado: persona con tratamiento completo, sin evidencia de fracaso, pero sin constancia de cultivos negativos consecutivos en los últimos meses de tratamiento.

Al comparar las condiciones de egreso del programa, se observó una proporción menor de pacientes fallecidos durante el tratamiento en Buenaventura (2/39; 5 %) frente a los demás municipios (9/60; 15 %). Entre los 11 pacientes fallecidos en tratamiento (mortalidad global = 11 %), tres eran diabéticos, cuatro estaban desnutridos y uno tenía HIV (cuadro 3). Igualmente, 15 (38 %) casos egresaron por pérdida de seguimiento en Buenaventura y 22 (35 %) en los demás municipios. El éxito del tratamiento (condición de curado o tratamiento terminado) se reportó en 22 casos (56 %) en Buenaventura frente a 29 (49 %) en los demás municipios).

**Cuadro 3:** Distribución de casos de pacientes fallecidos con tuberculosis resistente a rifampicina o multirresistente, por comorbilidad y sexo en el Valle del Cauca, Colombia, 2013-2016

Característica	Descripción	n	Buenaventura	Otros municipios del Valle del Cauca
Tipos de comorbilidad	Diabetes	3	1	2
	Desnutrición	4	1	3
	HIV	1	0	1
	Otras	1	0	1
	Sin comorbilidad	2	0	2
Sexo	Femenino	6	2	4
	Masculino	5	0	5

* Los datos corresponden a las comorbilidades identificadas y su distribución porcentual entre ambos grupos, por lo cual la suma dará 100 %.

** Distribución de casos con comorbilidades por sexo

## Discusión

En este estudio, se expone la evaluación de las diferencias sociodemográficas, clínicas y programáticas de pacientes con diagnóstico de tuberculosis RR o MDR entre Buenaventura y los demás municipios del departamento del Valle del Cauca. Este agrupamiento se realiza teniendo en cuenta que, por decisión administrativa, el esquema de tratamiento estandarizado y administrado empíricamente a los pacientes con dicha condición en Buenaventura difiere del convencional usado en Colombia.

Entre los pacientes con confirmación microbiológica de tuberculosis RR o MDR, una proporción importante no fue objeto del seguimiento programático (19/125; 15,2 %). Si bien esta proporción es menor que la estimada para el país en el 2015 (26 %), denota la necesidad de seguir trabajando en aumentar la disponibilidad de equipos con mayor capacidad para la gestión del monitoreo, el seguimiento y la evaluación de los casos. El seguimiento permite conocer, entre otras, la conversión bacteriológica, condición que contribuye a determinar la probabilidad de un resultado prometedor con la farmacoterapia instaurada y que ha sido asociada con resultados desfavorables del tratamiento en cohortes analizadas en Colombia ^8^ y en otras regiones del mundo ^9^.

Aunque, generalmente, las características sociodemográficas, de edad y de sexo de estas cohortes no difieren notablemente de las descritas global y localmente en la era de tratamiento de la tuberculosis RR o MDR con esquemas prolongados que incluían inyectables ^9^^,^^10^, vale la pena resaltar que en la cohorte de Buenaventura predominó el sexo femenino. Tampoco se encontraron proporciones de comorbilidades mayores de las estimadas previamente en la región y en el país ^8^^,^^10^. Sin embargo, dichas comorbilidades han influido en los resultados programáticos no favorables, como la mortalidad, datos coherentes con los encontrados en cohortes nacionales y de otras latitudes ^8^^,^^10^^,^^11^.

Aunque el objetivo de este estudio no fue describir las diferencias de la vigilancia epidemiológica entre las dos cohortes estudiadas, los hallazgos las exponen, dada la diferencia entre las proporciones de pacientes diagnosticados con diferentes metodologías para determinar los perfiles de resistencia y la prevalencia de los casos nuevos y los previamente tratados. Ciertamente, ya para el periodo del estudio, la OMS recomendaba la ejecución sistemática de una prueba molecular rápida para el diagnóstico de tuberculosis RR o MDR en pacientes con alto riesgo de farmacorresistencia ^12^, condición que aplicada en Buenaventura contribuyó al diagnóstico del 82 % (32/39) de dichos casos frente a solo un 23 % (26/43) en el resto de la región, donde se diagnosticaron los casos a partir de aislamientos en los que se les practicaron pruebas fenotípicas (12/60; 20,0 %) u otras pruebas moleculares (34/60; 56,7 %).

Otras variaciones en la vigilancia se identificaron mediante comportamientos de notificación irregular durante el periodo del estudio. Por ejemplo, en Buenaventura, en el 2015, disminuyó el número de casos detectados, mientras que en el resto del departamento aumentó. Para el año en mención, el Valle del Cauca, que tradicionalmente contribuía con cerca del 20 % de los casos nacionales, disminuyó su aporte al 15 %, probablemente por efecto de la reducción en la vigilancia de la resistencia en Buenaventura.

Este hecho evidencia la necesidad de mantener dicha vigilancia y complementarla con un aumento sostenido de las pruebas de sensibilidad a fármacos, como ya lo recomendaba la OMS en ese momento, lo cual fue adoptado por el programa nacional en el 2020 ^13^. De hecho, recientemente (2013 al 2019), ha aumentado el uso de GenXpert en Santiago de Cali, con una aplicación global de la prueba en pacientes con riesgo de multirresistencia del 46,8 %, lo que efectivamente ha contribuido al aumento de casos de tuberculosis RR o MDR detectados en esta ciudad ^14^. No se encontraron reportes de esta tendencia en Buenaventura o en el resto del departamento para dicho periodo, lo que evidencia la necesidad de estudios que arrojen nuevos datos sobre la implementación de estas medidas de vigilancia de la farmacorresistencia y su impacto en el control de la tuberculosis en diferentes entornos.

Uno de los intereses de este estudio fue evaluar si la frecuencia de RAFAS en Buenaventura era mayor en comparación con el resto de los municipios del Valle del Cauca y si esta diferencia también se observaba en los resultados programáticos, como la pérdida del seguimiento en los grupos comparados. Los hallazgos de este estudio demostraron que la frecuencia de RAFAS fue significativamente mayor en Buenaventura (p = 0,04); aunque la fuerza de asociación reportó un OR de 2,3 (IC_95%_: 0,993 - 5,568), el límite inferior del intervalo de confianza se aproximó al valor nulo, diluyendo la asociación posiblemente por el efecto del tamaño de la muestra. Esta asociación, aunque débil, puede explicarse por el uso combinado de etionamida y PAS que hace parte del esquema de Buenaventura y que otros estudios de la región, antes del tiempo de análisis de las presentes cohortes, ya asociaban con mayor número de RAFAS (gastrointestinales principalmente) y con mayores probabilidades de pérdida de seguimiento ^15^^,^^16^. Sin embargo, la pérdida de seguimiento, que globalmente fue del 36,4 % (36/99), solo fue ligeramente mayor en Buenaventura (15/39; 38,5 %) respecto al resto del departamento (21/60; 35 %).

Solo el uso de un número mayor de medicamentos y la mayor probabilidad de efectos adversos, especialmente gastrointestinales, no explica la elevada tasa de pérdida de seguimiento observada. De hecho, entre los 36 pacientes que abandonaron la terapia, 13 reportaron efectos adversos, ocho de índole gastrointestinal; sin embargo, solo tres eran de Buenaventura, el municipio con mayor reporte de efectos adversos, pero también con mayor proporción de casos objeto de monitoreo y gestión del tratamiento por parte del CERCET. En el estudio no se dispone de información suficiente para explicar el impacto de dicho comité en la prevención de la pérdida de seguimiento, asociada con el reporte oportuno y el manejo de eventos adversos. Sin embargo, casi la totalidad de los cambios en la terapia fueron hechos por dicho comité, la mayoría en pacientes de Buenaventura, lo que denota su potencial impacto y contribución a un mejor registro en el monitoreo y seguimiento de los casos, por lo cual su papel debe ser evaluado, socializado y, eventualmente, replicado en otros lugares.

En el mundo se ha descrito una mayor proporción de pérdida de seguimiento en pacientes con comorbilidades que en aquellos sin comorbilidades ^17^. En este estudio, fue evidente entre personas con HIV (4/8; 50 %), una proporción mucho mayor a la descrita en otras cohortes de la región de las Américas ^15^^,^^18^, pero que difiere de la estimada para pacientes con diabetes mellitus, cuya proporción fue similar a la de la cohorte general (5/14; 35,7 %). Otras cohortes analizadas en el país y las Américas han encontrado tasas elevadas de pérdida de seguimiento en personas con HIV o farmacodependientes, pero con proporciones globales más bajas en comparación con las analizadas en el Valle del Cauca ^8^^,^^18^.

La información registrada no permite evaluar componentes psicosociales asociados con la pérdida de seguimiento, aspecto relevante a la hora de realizar futuros análisis de cohortes, dado que en otros estudios se ha identificado la necesidad de intervenir los factores determinantes sociales para el control de la tuberculosis en la región, específicamente aquellos asociados con el cumplimiento del tratamiento ^19^, como recomienda actualmente la OMS, para promover un cuidado centrado en el paciente ^1^^,^^2^.

Entre los pacientes que no registraron pérdida de seguimiento, los de Buenaventura registraron mayor proporción de resultados favorables (22/39; 56,4 %) en comparación con el conjunto de los demás municipios (29/60; 48,3 %), pero las diferencias no fueron significativas. Esta proporción también fue mayor que la reportada a nivel nacional, que para el mismo periodo fue del 52 % en tuberculosis MDR ^10^, y similar al éxito de tratamiento reportado en las Américas en 2015 ^20^.

La tendencia en Buenaventura de presentar mejores resultados en cifras absolutas puede explicarse por el efecto de una mayor disponibilidad de herramientas diagnósticas moleculares durante el periodo analizado, lo que favorece un diagnóstico más temprano y un ingreso oportuno a la terapia para tuberculosis RR o MDR, tal como se ha reportado en revisiones sistemáticas y metaanálisis ^21^^,^^22^; en los demás municipios no se realizaba sistemáticamente una proporción de pruebas moleculares comparable con la de Buenaventura. Un hallazgo que refuerza esta premisa fue el menor tiempo transcurrido entre la práctica de la prueba de sensibilidad a fármacos y el inicio del tratamiento, (37 días en Buenaventura versus 75 días en los otros municipios).

Con el conocimiento previo de los patrones de resistencia ^5^^,^^23^ en Buenaventura, se ajustó el esquema de tratamiento por una mayor probabilidad de resistencia a fármacos de segunda línea, contenidos en el esquema IV, como kanamicina y levofloxacina. Esta resistencia no se evidenció con pruebas fenotípicas de sensibilidad porque en el 69 % (27/39) de los casos de tuberculosis MDR o RR no hubo disponibilidad de este tipo de pruebas para los medicamentos mencionados. Es posible que los cambios en el esquema terapéutico pudiesen haber tenido un beneficio potencial para prevenir la expansión de la infección y contribuir a los resultados de los tratamientos descritos. Los cambios farmacológicos en los pacientes de dicha cohorte incluyeron medicamentos que, posteriormente, mostraron mejores resultados terapéuticos según un metaanálisis ^24^. A su vez, los elementos descritos pueden haber contribuido a la ausencia de fracasos entre los casos tratados en Buenaventura y a que la mortalidad entre los mismos (2/39; 5,1 %) fuera inferior a la encontrada en el resto del departamento (9/60; 15 %) y a la estimada en el país para entonces (13 %) ^25^.

En años recientes, el manejo farmacológico de la tuberculosis MDR o RR ha cambiado, por lo que la OMS y la OPS promueven la incorporación de esquemas de tratamiento con medicamentos que ahora reportan menor toxicidad ^16^^,^^26^, permiten acortar la terapia ^27^ y resultan subsecuentemente menos costosos. Estos esquemas podrían mejorar la adhesión de los pacientes y los programas podrían obtener mayor proporción de resultados favorables. Desde el 2019 se recomienda la implementación de esquemas terapéuticos administrados totalmente por vía oral, que recientemente fueron acortados. No obstante este tipo de esquema corto está condicionado a pacientes nuevos, no expuestos a medicamentos de segunda línea y sin resistencia confirmada a fluoroquinolonas ^27^. Todo esto representa el gran reto de implementar dichas pruebas entre pacientes con diagnóstico de tuberculosis resistente a fármacos de primera línea.

En Colombia, con base en las recomendaciones de la OMS, el Programa Nacional de Control de Tuberculosis está en proceso de incorporar las nuevas directrices para el manejo terapéutico de los casos de tuberculosis MDR o RR. La implementación del esquema corto completamente oral, trae consigo dos claras ventajas para la región en términos de mejorar los resultados de tratamiento descritos: posiblemente mayor cumplimiento del tratamiento por la menor cantidad de medicamentos y la menor toxicidad asociada con los mismos; y mayor eficacia, dado que la bedaquilina no se ha usado antes en la región y, con pocas excepciones, el linezolid y la clofazimina, por lo cual la probabilidad de resistencia es muy baja y se disminuye cualquier riesgo de expansión mientras se dispone del resultado de la prueba de sensibilidad farmacológica.

Más recientemente, la OMS ha recomendado el uso de esquemas de seis meses para el tratamiento de tuberculosis MDR o RR. Su futura implementación en el programa departamental traería consigo beneficios adicionales en la terminación del tratamiento. Sin embargo, la introducción de nuevos esquemas y medicamentos requiere la preparación adecuada de la red de servicios para optimizar su uso, contribuir al control adecuado de la tuberculosis MDR o RR en la región y revisar las lecciones aprendidas.

Los hallazgos de esta investigación muestran las diferencias programáticas relacionadas con los esquemas de tratamiento para el manejo de casos de tuberculosis MDR o RR en los programas departamentales de control de tuberculosis del Valle del Cauca y el distrito de Buenaventura. Estas observaciones sirven como línea de base para la conducción de otras investigaciones operativas que permitan generar la evidencia necesaria para la toma de decisiones programáticas frente al diagnóstico, el seguimiento y el tratamiento, necesarias para optimizar las medidas de prevención y control de la tuberculosis MDR. Asimismo, contribuyen a identificar las potenciales debilidades y fortalezas de los programas y dan paso a la implementación de las actuales recomendaciones -en términos de vigilancia de la farmacorresistencia y su control farmacológico- hechas por la OMS y adaptadas recientemente por el programa nacional ^13^.

El estudio evidenció debilidades en la vigilancia de la farmacorresistencia por su heterogeneidad. Por tanto, es necesario establecer cómo se hace el diagnóstico de tuberculosis y su confirmación bacteriológica, y cómo ello afecta los resultados programáticos. Así, el programa regional podría enfocar sus esfuerzos hacia la identificación temprana de farmacorresistencia en todos los pacientes ingresados al programa y su direccionamiento hacia la mejor terapia farmacológica disponible. Dada la fortaleza de disponer de una red diagnóstica con acceso a pruebas moleculares y convencionales para evaluar la resistencia, se debe avanzar en el uso de algoritmos que favorezcan la universalidad de las pruebas entre los que serán tratados dentro del programa para el control de la tuberculosis.

En este estudio se presentan varias limitaciones. La primera es que es un análisis de registros históricos de fuentes secundarias, por lo cual el carácter retrospectivo de los datos puede comprometer la calidad de la información y presentar un subregistro, principalmente de los RAFAS y los seguimientos bacteriológicos, pues la mayoría de las tarjetas de tratamiento de categoría IV estaban impresas y fueron diligenciadas manualmente por el personal de atención, quien no realizaba la totalidad de los registros (probablemente por la complejidad y extensión del formato).

La segunda limitación es que el alcance de este estudio no permite determinar factores asociados con los resultados favorables, ni se hizo un análisis diferencial por historia de tratamiento, tipo de prueba diagnóstica de la condición resistente, ni por cada medicamento.

La tercera es que no se calculó el tamaño de la muestra, dado que se incluyeron todos los registros disponibles; por lo tanto, los resultados brindan un panorama real del comportamiento del evento, pero el número limitado de los registros disponibles representa intervalos de confianza amplios que pueden comprometer la precisión de los datos. Siendo un estudio retrospectivo de cohorte, no se utilizó como medida de asociación el riesgo relativo sino la razón de probabilidades, por considerar que es más versátil y que con una adecuada interpretación puede usarse en cualquier diseño. Además, para eventos como la tuberculosis multirresistente en estas circunstancias, con una frecuencia menor del 10 %, los resultados con ambas medidas pueden ser similares y, de requerirse un ajuste multivariado, la razón de probabilidades se puede modelar con regresión logística, lo que no permite el riesgo relativo.

Finalmente, el estudio permite concluir que, en un mismo programa departamental de control de tuberculosis, como el del Valle del Cauca, se presentan algunas divergencias demográficas, clínicas y de resultados programáticos que ameritan abordajes diferenciales, toda vez que la tuberculosis farmacorresistente continúa siendo un obstáculo para el control de la tuberculosis en el Valle del Cauca y en el distrito de Buenaventura. La identificación temprana y el óptimo manejo integral de los casos multirresistentes deben seguir siendo parte de las prioridades del programa.

Por otro lado, se evidenció que, aunque el esquema de tratamiento de Buenaventura tendía a presentar mayor proporción de RAFAS, cuando se cuenta con un programa fortalecido que hace seguimiento permanente a los pacientes, con acceso a pruebas diagnósticas y a comités de expertos como el CERCET, los resultados son mejores, lo que refuerza la importancia de las estrategias mencionadas como un derrotero para todos los programas de control de la tuberculosis.

A manera de recomendación, a pesar de los resultados favorables del tratamiento, comparables a nivel nacional y regional, las tasas elevadas de pérdida de seguimiento disminuyen ostensiblemente la probabilidad de que los resultados sean mejores. La tuberculosis continúa siendo un problema social que requiere un abordaje integral.
